# Tissue and Serum microRNAs in the Kras^G12D^ Transgenic Animal Model and in Patients with Pancreatic Cancer

**DOI:** 10.1371/journal.pone.0020687

**Published:** 2011-06-27

**Authors:** Joseph J. LaConti, Narayan Shivapurkar, Anju Preet, Anne Deslattes Mays, Ivana Peran, Sung Eun Kim, John L. Marshall, Anna T. Riegel, Anton Wellstein

**Affiliations:** Lombardi Cancer Center, Georgetown University, Washington, D.C., United States of America; University of Medicine and Dentistry of New Jersey, United States of America

## Abstract

microRNAs (miRs) modulate the expression levels of mRNAs and proteins and can thus contribute to cancer initiation and progression. In addition to their intracelluar function, miRs are released from cells and shed into the circulation. We postulated that circulating miRs could provide insight into pathways altered during cancer progression and may indicate responses to treatment. Here we focus on pancreatic cancer malignant progression. We report that changes in miR expression patterns during progression of normal tissues to invasive pancreatic adenocarcinoma in the p48-Cre/LSL-Kras^G12D^ mouse model mirrors the miR changes observed in human pancreatic cancer tissues. miR-148a/b and miR-375 expression were found decreased whereas miR-10, miR-21, miR-100 and miR-155 were increased when comparing normal tissues, premalignant lesions and invasive carcinoma in the mouse model. Predicted target mRNAs FGFR1 (miR-10) and MLH1 (miR-155) were found downregulated. Quantitation of nine microRNAs in plasma samples from patients distinguished pancreatic cancers from other cancers as well as non-cancerous pancreatic disease. Finally, gemcitabine treatment of control animals and p48-Cre/LSL-Kras^G12D^ animals with pancreatic cancer caused distinct and up to 60-fold changes in circulating miRs that indicate differential drug effects on normal and cancer tissues. These findings support the significance of detecting miRs in the circulation and suggests that circulating miRs could serve as indicators of drug response.

## Introduction

microRNAs (miRNAs or miRs) are small, non-coding RNAs that play a significant role in controlling the activities of cellular pathways both in physiology and pathology (see e.g. [1]). The distinct function of miRs in different cancers has become more obvious over the past years [2], [3], and many studies show that miR signatures can be used to distinguish different cancers [4], [5], [6], [7] prognoses [8], [9], [10], [11], [12], [13] or reveal potential targets [14] as well as altered signaling pathways [15]. Most surprisingly, a comparison of miR and mRNA profiles of primary and metastatic cancer lesions showed that miRs provided a more reliable and distinctive signature than mRNAs and found that miR signatures were superior to mRNAs in identifying the organ source of metastases of unknown origin [16], [17]. Beyond these analyses of normal and diseased tissues, more recent reports have shown that miR species can be detected in the circulation [18] and suggested that analysis of serum samples for defined miR species could be used to identify patients with cancers [19], [20], [21], [22], [23], [24], [25] as well as other diseases such as cardiac disease [26], [27], [28], [29], [30] or diabetes mellitus [31].

Sequences of miRs are frequently conserved across species and we speculated that analysis of miRs in a well-defined animal model could inform studies with patient samples. We were particularly interested to evaluate whether this could be translated into the detection and quantitation of miRs in the circulation because that might ultimately reveal activated or altered disease pathways based on the analysis of a blood sample rather than the analysis of diseased tissue specimen [32]. In addition, treatments will likely impact miR patterns in the circulation and these patterns may well be useful in establishing signatures of drug effects.

Here we focused on pancreatic cancer, that was diagnosed in 43,140 patients in 2010. Pancreatic cancer is a fatal disease with a 5-year survival rate of only 6% [33]. This poor outcome is due to late detection as well as a lack of effective therapies [34]. To identify informative miRs, we used a genetically engineered mouse model, the p48-Cre/LSL-Kras^G12D^ model, that was first described by Hingorani *et al.* [35]. This model faithfully reproduces the malignant progression seen in human PDAC development [34], [35] and numerous studies with this model narrowed down the cells of origin of PDAC [36] and showed the contribution of different driver genes [37], [38], [39] that control the biology and progression of this disease [40]. We used tissues harvested at different stages of malignant progression from this mouse model to evaluate a panel of miRs that had been shown to be up- or down-regulated in human pancreatic cancer tissues and had been reviewed and compiled recently by Seux and colleagues [41]. This analysis was then followed by quantitation of miRs in the circulation of patients with pancreatic and other cancers or controls and we found miR expression patterns that distinguished between the different groups. miR expression patterns in serum from experimental animals paralleled the findings in patients. Finally, treatment of animals with the anti-cancer drug Gemcitabine that is approved for first line therapy of pancreatic cancer [42], showed a distinct pattern change in miR levels in the circulation of animals with pancreatic cancer versus controls.

## Results

### microRNA expression in pancreatic tissues during Kras^G12D^-induced malignant progression

A panel of miRs consistently up- or down-regulated across different studies in human pancreatic cancer tissues relative to normal pancreatic tissues was selected from literature and data base searches (see Table S1; Refs [7], [41], [43], [44], [45]). For this miR panel we established quantitative RT-PCR detection [46] because we expected a wide range of miR concentrations when comparing tissue extracts versus blood samples or across human and murine samples.

**Figure 1:**
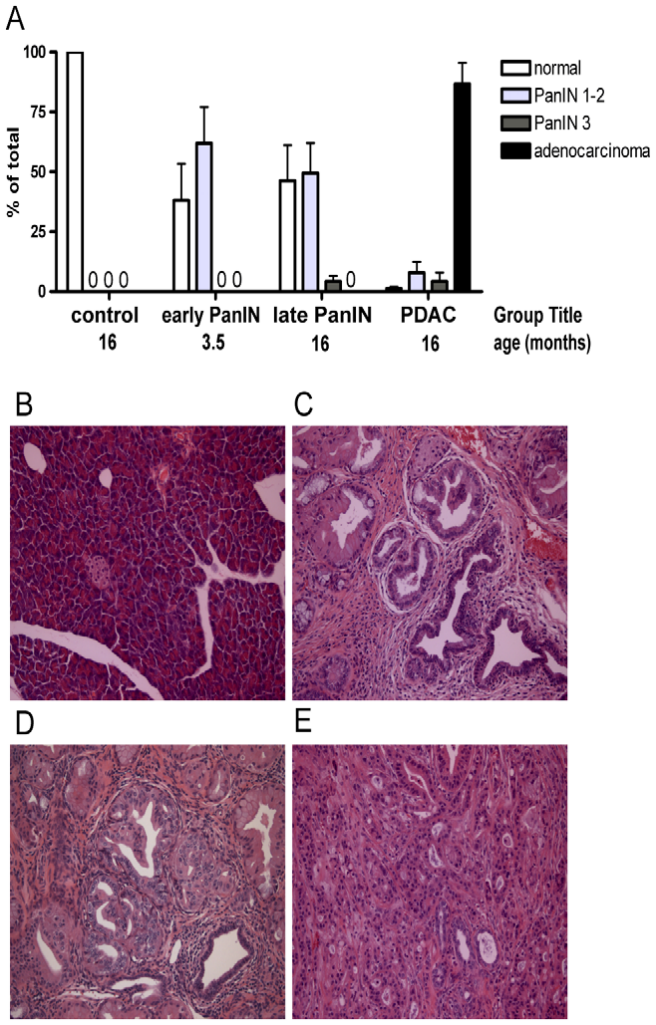
Malignant progression of pancreatic duct epithelia in p48-Cre/LSL-Kras^G12D^ mice. (A) Quantitation of histopathologic alterations in the pancreata of controls or p48-Cre/Kras^G12D^ mice. The samples were separated into controls, early stage dysplastic lesions (PanIN-1 and -2), late stage dysplastic lesions (PanIN-3 present) and invasive pancreatic duct adenocarcinoma (PDAC). (B to E) Representative histopathology images from each of the groups: (B) normal pancreas, (C) PanIN-1 and -2 (early), (D) PanIN-3 (late) and (E) PDAC. Mean ± standard error of the % of the pancreatic tissue with the respective lesions is shown (n = 3 animals for each group). 0, not detected.

Mouse pancreatic tissue samples were harvested at different ages from the p48-Cre/LSL-Kras^G12D^ mouse model. Pancreatic duct epithelia in these animals progress through early and late dysplastic lesions, PanIN ( = Pancreatic in situ carcinoma) over the period of several months to invasive cancer and thus mimic malignant progression of the human disease [34], [35]. Each tissue sample harvested was staged by a histologic analysis of pancreatic ductal changes (Figure 1A). Control tissues contained 100% normal ducts (Figure 1B). Pancreata from younger mice (Figure 1C) contained more than 50% of ducts with early stage dysplastic lesions (PanIN-1 or -2). Pancreata from older mice (Figure 1D) contained ∼10% of ducts with late stage dysplastic lesions (PanIN-3) in addition to ∼50% of ducts with PanIN-1 or -2. PDAC tissues contained mostly invasive adenocarcinoma (Figure 1E).

**Figure 2:**
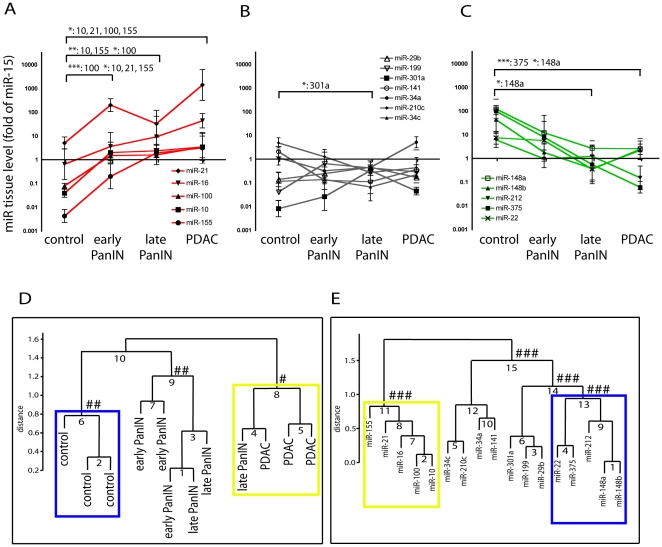
miR expression in mouse pancreatic tissues during malignant progression. (A–C) Expression levels of individual miRs in control and pancreatic tissues at different stages of malignant transformation. The miRs levels were grouped as increasing (A), steady (B), or decreasing (C) based on a comparison of the levels in each group (n = 3 per group). Mean ± standard error is shown for each miR expression. (D) Hierarchical clustering of mouse tissues based on miR expression. Distinct groups are indicated by the blue and the yellow box. (E) Hierarchical clustering of miRs based on their expression levels. miRs that were expressed at high levels in control (blue box) versus PDAC tissues (yellow box) are indicated. * p<0.05, ** p<0.01, *** p<0.001; control vs. early PanIN, late PanIN, or PDAC. #: au >0.85 and p = 0.06, ##: au >0.85 and p<0.05, ###: au >0.90 and p<0.01. (au, approximately unbiased probability).

Analysis of the expression of individual miRs showed three major trends (Figure 2A–C): First, expression of miR-10, miR-16, miR-21, miR-100 and miR-155 increased in the early PanIN lesions relative to control, and maintained high expression in the late PanIN and adenocarcinoma tissues. (Figure 2A) Second, miR-22, miR-148a/b, miR-212, and miR-375 were highly expressed in control tissues and their expression was reduced in PanIN (Figure 2C) as well as in adenocarcinoma tissues. Third, expression of miR-29b, miR-34a/c, miR-141, miR-199, miR-210c and miR-301a did not change significantly during malignant progression (Figure 2B).

### Distinct clustering of miRs and of pancreatic tissues with different disease stages

An unsupervised clustering of the mouse tissues based on their miR expression levels identified three distinct groups (Figure 2D). The control tissues separated from all other tissues in a grouping of their own (blue box). Five of six tissues classified as in situ lesions (PanIN) were clustered together in a second group. Invasive adenocarcinoma and one of the late PanIN tissues segregated into a further cluster (yellow box). Thus, the set of miRs analyzed here is sufficient to distinguish the different stages of mutant Kras-induced pancreatic malignant progression.

Unsupervised clustering of the individual miRs was performed to determine which miRs behave in parallel and can thus serve as common signatures coincident with disease stage (Figure 2E). One group (yellow box) contained those miRs that showed the highest expression in the adenocarcinoma tissues and the lowest in the control tissues. A separate group (blue box) showed a reciprocal miR expression pattern, with the highest levels in control tissues and the lowest levels in adenocarcinoma. These groupings suggest that a subset of miRs can define a tissue's classification corroborating earlier work from others with different human cancer samples [16], [17].

A comparison of the findings in the mouse model (Figure 1 & 2) with published studies in human pancreatic cancers shows the same qualitative changes for most of twelve miRs analyzed in both settings (Table S1; Refs [7], [43], [44], [45]): Seven miRs upregulated in cancer versus normal tissues in the mouse model were also upregulated in human cancers. Of five miRs found downregulated in the mouse model, four were also downregulated or showed no change in studies with human specimen. Only miR-212 was upregulated in human and downregulated in mouse PDAC samples. It is tempting to speculate that the discordance of miR-212 between human and mouse PDAC samples may indicate species differences of epithelial-stroma interactions during malignant progression [47]. Overall, the close coincidence of miR changes in malignant pancreatic tissues across species and across different studies suggests that clinical pancreatic adenocarcinoma is represented well by the p48-Cre/LSL-Kras^G12D^ animal model.

### Expression of miR target genes and miRs in mouse pancreatic tissues

**Figure 3:**
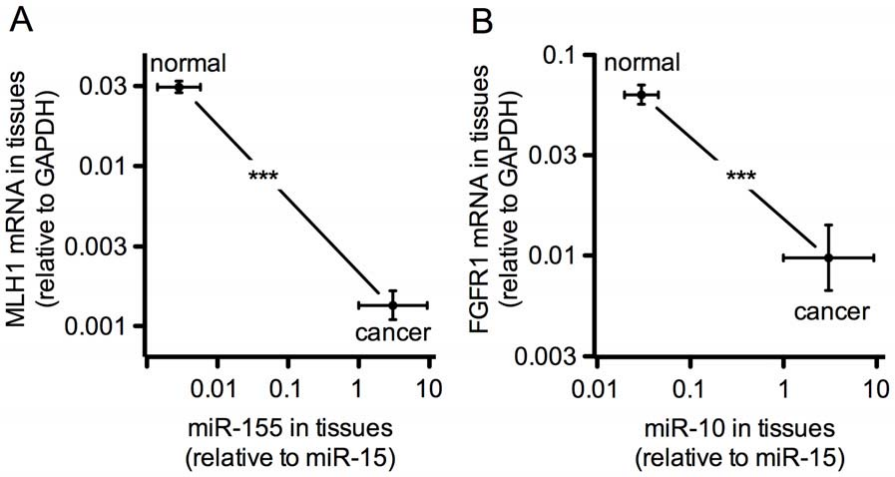
Correlation between the expression of predicted mRNA target genes and miRs in mouse normal and pancreatic cancer tissues (PDAC). Tissues from p48-Cre/Kras^G12D^ mice with invasive pancreatic ductal adenocarcinoma (PDAC) and normal pancreata were analyzed for expression of miR-10 and miR-155 relative to the respective candidate target mRNAs, FGFR1 and MLH1 using quantitative RT-PCR. Mean ± SEM of n = 3 in each group; ***, P<0.001 normal versus cancer.

Recent studies have shown that the predominant activity of miRs (84%) is their impact on target mRNA steady-state levels [48]. To assss this in the mouse model, we identified candidate mRNA targets from an unbiased list of under-expressed mRNAs in human pancreatic cancers and matched these with the miR panel studied here (Table S3). The set of matching genes downregulated in pancreatic cancer contains MLH1 as a predicted target for miR-155, and FGFR1 as a target of miR-10. In a comparison of normal and cancer tissues harvested from the mouse models mRNA expression of MLH1 and of FGFR1 showed a significant, inverse relationship miR-155 and miR-10 respectively (Figure 3).

### Gemcitabine treatment effect on circulating miRs in the animal model

**Figure 4:**
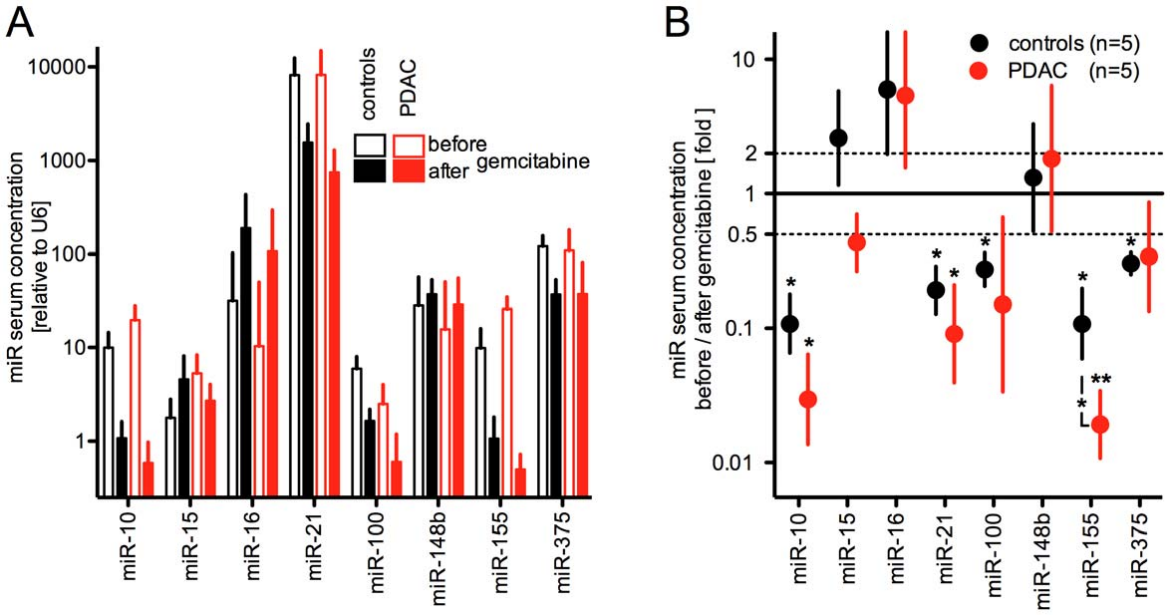
Effect of Gemcitabine treatment on serum miR levels of p48-Cre/LSL-Kras^G12D^ mice with PDAC (red; n = 5) or age-matched control mice (black; n = 5). (A) miR-levels in serum samples harvested before (open bars) and after a one week treatment with Gemcitabine (5 doses of 40 mg/kg). (B) Ratio of serum concentrations before / after Gemcitabine treatment. The dotted line indicates a two-fold difference. *, p<0.05; **, p<0.01.

The presence of diseased tissues may be indicated by altered miR concentraions in the circulation (see Introduction). As a logical extension, miR concentrations in the circulation could also serve as easily accessible markers of treatment efficacy and even indicate pathways altered by a given treatment. We tested this hypothesis in the PDAC mouse model relative to control animals without cancer. Gemcitabine is a first-line drug used in the treatment of patients with pancreatic cancer and was administered for one week to animals with PDAC and to age-matched control animals. The dose and treatment schedule were adapted from other studies that had shown efficacy over a longer treatment period [49], [50]. A small blood sample (<0.1 ml ) was drawn before initiation of treatment to compare miR serum levels before and after treatment across these two groups of animals. The presence of PDAC in the p48-Cre/Kras^G12D^ animals was confirmed by histological analysis of pancreatic tissues at the end of the study. We selected six miRs that were found upregulated and two that were down regulated in PDAC tissues relative to controls (see Figure 2). A>10,000-fold concentration range of these eight miRs was found in the circulation of animals (Figure 4A). Before treatment (Figure 4A, open bars), serum levels of miR-10 and miR-155 were elevated >2-fold (p<0.05) in the PDAC (red) versus control group (black). In contrast, serum levels of miR-21, miR-148b and miR-375 where indistinguishable between the groups. Gemcitabine treatment (Figure 4A, filled bars) reduced serum levels of miR-10, miR-21 and miR-155 in animals with PDAC and in controls by 6- to 60-fold (p<0.05 to <0.01; Figure 4B). Serum levels of miR-100 and miR-375 were reduced by >2-fold after the treatment though only the controls showed statistically significant differences (p<0.05). miR-148b serum levels were not altered by the treatment and miR-16 levels increased >5-fold after treatment. It is noteworthy that Gemcitabine treatment of animals with PDAC reduced serum levels of miR-21, miR-10 and miR-155 by an additional 2-, 3- and 6-fold below the reduction seen in control animals, although only the miR-155 reached statistical significance in the comparison of PDAC and control (Figure 4B; p<0.05). This data suggests that monitoring appropriate miRs in the circulation may distinguish drug effects on diseased tissues from the drug effects on the healthy non-target tissues.

### miRs in the circulation of patients with pancreatic and other cancers

**Figure 5:**
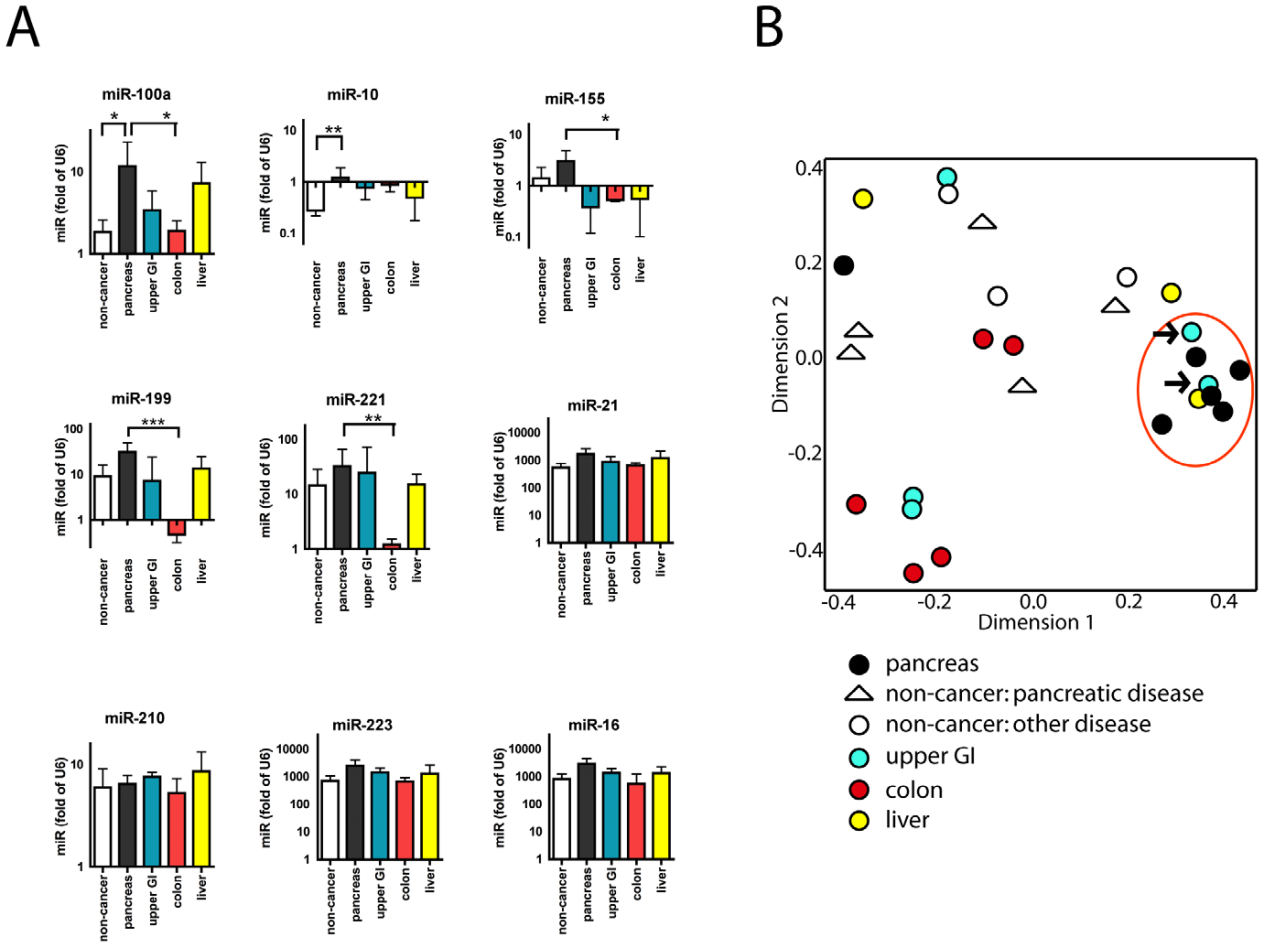
miRs detected in human plasma samples. Samples were from pancreatic cancer patients, non-cancer controls, and patients with other GI cancers. (A) Concentrations of nine miRs detected in the circulation show individual differences between patients groups. Note the different ranges of the scales on the Y axes. (B) Unsupervised random forest analysis comparing pancreatic cancer (black circles) versus non-cancer controls with pancreatic disease (white triangle), non-cancer controls without pancreatic disease (white circles), upper GI cancer (blue circles), colon cancers (red circles), and liver cancers (yellow circles). Circled in red are the majority of the pancreatic cancers. Arrows indicate two specimen from patients with duodenal cancer. * p<0.05, ** p<0.01, *** p<0.001. Patient characteristics are provided in Table S2.

Nine different miRs were isolated and quantitated from plasma samples of patients with pancreatic cancers, other gastrointestinal cancers, and non-cancer controls. The patient diagnoses are summarized in Table S2. miR-100a and miR-10 were significantly increased in the pancreatic cancer patients compared to non-cancer controls while a number of the other miRs (miR-16, 21, 155, 199, 221, and 223) showed a trend of increased expression that did not reach statistical significance (Figure 5A). Another subset of miRs showed significant expression differences between pancreatic cancer and colon cancer patients, but not relative to patients with other gastrointestinal cancers. The expression patterns of the different circulating miRs suggest that some are best at distinguishing between cancer and non-cancer patients whilst others best distinguish the diseased organs.

In an unsupervised random forest analysis that considered the expression of all nine miRs isolated from the circulation, five of six pancreatic cancer patients grouped together in a separate group from the majority of other patients (Figure 5B). This confirms that the combined expression pattern of these nine miRs in the circulation was sufficient to identify patients with pancreatic cancer as separate from patients with other GI cancers and controls. Of note were two specimens from patients with duodenal neoplasms (arrows) that grouped closest to pancreatic cancer patients, possibly due to pancreatic involvement undetected at the time of sampling. Also, samples from patients with non-cancerous pancreatic disease and non-cancer controls grouped together indicating that the panel of miR expression is specific for pancreatic cancer rather than any disease originating from the pancreas.

## Discussion

The mutant Kras^G12D^–driven pancreatic cancer model has been well characterized at numerous molecular and biological levels [35], [36], [37], [38], [39]. Our analysis of tissue samples shows that some miR changes associated with invasive pancreatic cancer are already apparent during the early stages of the disease (Figure 2). More surprising was the extent to which changes in miR expression in the animal model mimicked miR expression changes observed in human pancreatic cancer (see Table S1). Indeed, a subset of miRs that includes miR-10 and miR-155 were upregulated in pancreatic cancer tissues of patients and mice as well as in the respective blood samples. Thus, this study provides evidence that the inter-species similarity of miR expression in the context of pancreatic cancer is relatively conserved, very likely due to mutant Kras as a major initiator of this malignancy [42].

The comparative analysis of miR expression during malignant progression in the mouse model allows us to draw some conclusions about relevant miRs in the circulation that can indicate the presence of precursor lesions. Habbe et al. [51] reported on the expression levels of miRs in human intraductal papillary mucinous neoplasms (IPMN) tissues, and concluded that miR-155 is upregulated and a possible tissue biomarker of pre-invasive disease. We found miR-155 to be upregulated in the sets that contain PanIN lesions in the mouse model and also found miR-155 upregulated in plasma samples from pancreatic cancer patients. Other studies evaluating circulating miR-21, miR-210, miR-155 and miR-196a in different sets of pancreatic cancer patients have drawn similar conclusions on the diagnostic potential of miRs [52]. IPMN, mucinous cystic neoplasm (MCN), and PanIN represent three known precursor lesions of PDAC. These three types of premalignancies have many genetic and pathologic similarities, but also some features that allow to differentiate them [53]. Our findings support the hypothesis that plasma levels of miR-155 may indeed represent a biomarker indicating the presence of PanIN lesions.

Similar miR changes in tissues and in the circulation suggest to us that miRs are released from the diseased tissues in a continuous manner possibly via exosomes [54], [55] although there may be specific release mechanisms that may favor some miRs over others [56]. Here we mostly focused on miRs that are elevated in diseased tissues rather than those whose expression is reduced or lost. We reasoned that a loss of a given miR will only impact on steady-state levels in the circulation if the diseased organ is the major source of the miR present in the circulation. E.g. miR-148a/b and miR-375 are downregulated very strongly in pancreatic cancer relative to normal pancreatic tissues. miR-375 has been shown to play a major role in pancreatic islet development [57], and function as well as in the maintenance of glucose homeostasis [58], [59] and it is noteworthy that one early symptom of PDAC can be adult onset diabetes mellitus. miR-148 can repress expression of DNMT3b through a region in its coding sequence [60] and can thus impact DNA repair mechanisms.

In spite of a>100-fold reduction of miR-375 and miR-148a/b during malignant transformation of pancreatic tissues, it is striking that their serum levels are not reduced in animals with PDAC (see Figure 2C and 4A). This suggests that pancreatic contribution to their serum levels is only small. This is likely also true for miR-21 where increases in tissue levels of >100-fold during pancreatic malignant transformation in the animal model are not reflected in increased serum levels (see Figure 2A and 4A). In contrast, miR-10 and miR-155 levels in serum are increased during pancreatic malignant transformation in mice and in patients supporting the notion that the diseased organ is a significant contributor to serum levels of these miRs (see Figure 4A and 5A).

Recent studies from the Bartel laboratory have demonstrated that the predominant activity of miRs is to decrease target mRNA levels and found that over 84% of miR effects on protein production are due to this depletion of target mRNA [48]. Thus, miRs that are upregulated in the circulation of diseased subjects may coincide with reduced levels of target mRNAs in the diseased tissue of origin. We further hypothesized that miRs found to be increased in the circulation of patients might be present at much higher levels in the diseased tisses due to the dilution upon their shedding into the blood stream. We identified candidate mRNA targets and an unbiased list of under-expressed mRNAs from pancreatic cancer versus normal tissues compiled from different studies returned 154 mRNAs that could be cancer relevant miR targets. These were matched with the miR panel studied here (Table S3).

The set of genes returned from this analysis contains *MLH1* predicted as a target for miR-155. Indeed, overexpression of miR-155 in cell lines resulted in down-regulation of hMSH2, hMSH6, and hMLH1. Also, an inverse correlation between expression levels of miR-155 and MLH1 or MSH2 proteins was reported for human colorectal cancers [61]. MLH1 is a mis-match repair protein that contributes to the accumulation of genetic errors in the context of familial pancreatic cancer and some sporadic cases [34]. Its mRNA was found downregulated in pancreatic cancer samples and a fraction of the loss of MLH1 mRNA expression in pancreatic cancers has been attributed to promoter hypermethylation [62]. We observed a significant inverse relationship between the expression of miR-155 and MLH1 mRNA in the comparison of normal and cancer tissues (Figure 3A). In addition, FGFR1 mRNA was found downregulated in human pancreatic adenocarcinoma (Table S3) and we observed a significant downregulation in the mouse PDAC model relative to normal tissues (Figure 3B). These findings with miR-10 and miR-155 and their predicted target mRNAs MLH1 and FGFR1 support the notion of a potential regulatory function of these miRs during malignant progression.

In a further series of animal studies with potential direct clinical application, we tested whether miRs could indicate drug efficacy. The concentration range of circulating miRs monitored in this experiments is >10,000-fold and the impact of the drug treatment was unrelated with the pre-treatment concentration of the eight miRs monitored (Figure 4A). After Gemcitabine treatment miR-16 was found increased in the serum by 5-fold in PDAC as well as control animals. miR-16 expression is associated with apoptosis [63], growth suppression through p53 [64] and tumor suppression [65]. The increase of this miR-16 in the circulation cancer and control animals matches with the cytotoxic activity of the drug on healthy tissues. In contrast with this increase, miR-148b serum levels were not affected after the drug treatment and serum miR-10 and miR-155 were reduced the most (30- and 60-fold) after the Gemcitabine treatment. These two miRs had been found elevated in the serum of PDAC animals relative to controls before initiation of the drug treatment. Furthermore, serum levels of miR-10 and miR-155 in treated animals with PDAC dropped below the serum levels of treated control animals (Figure 4A & B) suggesting them as potential indicators of tumor specific effects of the treatment. Overall, the findings in this experimental setting support the concept of a pancreatic cancer selective efficacy of Gemcitabine though effects on the homeostasis of other healthy tissues also became apparent.

### Conclusion

miR changes in tissues and the circulation show remarkable similarities between pancreatic cancer in patients and the p48-Cre/Kras^G12D^ mouse model of the disease. Beyond the mimcry of human molecular pathology in the mouse model, the signature miRs identified here may also serve as informative indicators of drug efficacy in the development of desperately needed single agent or combination therapies [42] of this devastating disease.

## Materials and Methods

### Mouse tissue analysis

Animal study protocols were approved by the Georgetown University Animal Care and Use Committee (GUACUC #08-028). The p48-Cre/LSL-Kras^G12D^ mouse model has described previously [35]. In the control, late PanIN and adenocarcinoma groups, mice were sacrificed at 16 months of age. Controls lacked either the KRAS^G12D^ or the p48-CRE allele. For the early PanIN group, mice at one month of age were treated with caerulin and sacrificed at four months of age following an established protocol [66]. Pancreata were bisected from tail to head with one half fixed in formalin and the other half frozen in liquid nitrogen. A pathologist scored the highest PanIN grade per lobule of all lobules counted in a representative H&E stained slide of each mouse's pancreas [35]. “Normal” includes any normal and reactive ductal change. PanIN-1 and -2 were combined into a single category of “early” lesions while tissues with PanIN-3 were included in a separate category of “late” lesions due to the high likelihood of malignant progression.

### miR and mRNA expression in mouse pancreatic tissue

Total RNA was extracted from tissues using the TRIZOL reagent (Invitrogen, Carlsbad, CA) as described by the manufacturer. miRs were further isolated from the total RNA using a miR isolation kit (SA Biosciences, Frederick, MD). The miR was converted to cDNA using polyA tailing followed by universal priming with miR First Strand Kit and quantitated using pre-designed miR specific qPCR (SA Biosciences, Frederick, MD) on an ABI 7900 HT Real-Time PCR system (Applied Biosystems, Foster City, CA). mRNA quantitation was described in [46]. In brief, cDNA was synthesized using the total RNA extracted and the iScript cDNA Synthesis Kit, according to the manufacturer's protocol (Bio-Rad Laboratories). qRT-PCR was performed using iQ SYBR Green Supermix (Bio-Rad Laboratories) on an iCycler (BioRad) with: 95°C for 3 min followed by 40 cycles (95°C for 20 sec, 60°C for 30 sec and 72°C for 40 sec) and melting curve step (95°C for 1 min, 55°C for 1 min, increased temperature gradient from 50°C by 0.5°C each 10 sec in the following 80 cycles). MLH1 forward primer: GCGGCACCCACTTCCAGTCC; reverse: CGGAGAGTCTCATGGCACCGC. FGFR1 forward primer: GTAGCTCCCTACTGGACATCC; reverse primer: GCATAGCGAACCTTGTAGCCTC.

### miR detection and quantitation in human and mouse blood samples

Human blood samples were obtained from the biorepository of the Lombardi Cancer Center that collects anonymized specimen from cancer and non-cancer patients for research purposes. The data were analyzed anonymously. Mouse blood (<0.1 ml) was collected via submandibular bleeding using a lancet [67]. Serum or plasma samples were mixed at a ratio of 1∶10 with Qiazol lysis reagent and vortexed. The lysate was extracted with CHCl3 and the aqueous phase was further processed for total RNA using the miRNeasy Mini kit (Qiagen, Valencia, CA) and enriched for miRNA using the RT2 qPCR-Grade miRNA Isolation Kit, MA-01 (SABiosciences).

### Gemcitabine treatment of animals

Gemcitabine was obtained from the hospital pharmacy and administered to 22–23 month old p48-Cre/Kras^G12D^ or age matched control animals at 40 mg/kg in 5 doses over the course of one week. This dose of 200 mg/kg/week was based on Refs. [49], [50]. Blood was drawn before initiation of treatment (<0.1 ml ) and one day after the last dose. The presence of PDAC was confirmed in the p48-Cre/Kras^G12D^ by postmortem histological analysis.

### Data analysis

The data processing methods were encoded in R (http://www.r-project.org). Hierarchical clustering was performed based on the mean centered and scaled miR expression levels. The clustering methods used are available as *pvclust* [68] and *sbfit* [69]. These methods allow for the calculation of significance between the hierarchical clusters. The figure legends provide the approximately unbiased probability (au) metrics along with P-values determining the significance of the cluster separation. A random forest classification algorithm [70] was applied to the data sets in an unsupervised mode, with the number of trees set at 2000 and two or three variables. Prism 5.0 (Graphpad-Software was used for other tests and display of the data. Mean ± SEM are depicted unless indicated otherwise.

### In silico miR target gene search

Four microarry studies from the Oncomine data base (http://www.oncomine.org/) were chosen because they represented a comparison of mRNA changes in normal or control tissues versus pancreatic ductal adenocarcinoma [71], [72], [73], [74]. Initially, a list of all genes that were found downregulated in cancer tissues was generated assuming that a miR that targets a given gene could be reducing its steady state expression levels. Over 2500 genes were reported as downregulated amongst the four studies chosen. This list was shortened to 154 genes by applying the filter ‘Cancer Gene Census – all causal cancer genes’. If a gene was downregulated significantly (p<0.05) in one of the studies, it was searched for possible predicted target miRs using three databases: http://microrna.sanger.ac.uk/cgi-bin/targets/v5/search.pl, http://pictar.mdc-berlin.de/cgi-bin/PicTar_vertebrate.cgi, http://www.targetscan.org/ The list we report represents a possible target for the miR based on target stringency determined by each database, with the Sanger data base receiving the highest priority. Table S3 summarizes the findings.

## Supporting Information

Table S1 **miR levels in mouse and human pancreatic adenocarcinoma relative to normal tissues.** Tissues from the p48-Cre/LSL-Kras^G12D^ mouse model (ranked from the highest to lowest levels) are shown in parallel with published data from clinical samples. The antilog_2_ of the measurements is provided with 0 indicating no change and values below -0.5 or above +0.5 considered as biologically relevant changes relative to controls. ^a^ compared to IPMN; ^b^ miR-210 not miR-210c; NR, no report of this miR in human samples; * p<0.05, *** p<0.001 vs. control.
(DOC)

Table S2 **Patient diagnoses and characteristics for the plasma sample set analyzed.**
(DOC)

Table S3 **mRNA expression of miR target genes in pancreatic adenocarcinoma.** mRNA expression levels were obtained from published studies that compared normal and cancer tissues. Targets for those miRs found upregulated in the circulation of pancreatic cancer patients were derived from an *in silico* analysis (see Methods). n, number of samples per group. * p<0.05, ** p<0.01, *** p<0.001 downregulation in cancer versus control tissues.
(DOC)
