# Supplementary material for: Tissue and Serum microRNAs in the KrasG12D Transgenic Animal Model and in Patients with Pancreatic Cancer
Source: PLoS One. 2011 Jun 27;6(6):e20687. doi: 10.1371/journal.pone.0020687 (PMC3124473; doi:10.1371/journal.pone.0020687)
Supplement: Table S1 — miR levels in mouse and human pancreatic adenocarcinoma relative to normal tissues. Tissues from the p48-Cre/LSL-KrasG12D mouse model (ranked from the highest to lowest levels) are shown in parallel with published data from clinical samples. The antilog2 of the measurements is provided with 0 indicating no change and values below -0.5 or above +0.5 considered as biologically relevant changes relative to controls. a compared to IPMN; b miR-210 not miR-210c; NR, no report of this miR in human samples; * p<0.05, *** p<0.001 vs. control. (DOC) [file pone.0020687.s001.doc]

| miR | Mouse | Human | Reference |
| --- | --- | --- | --- |
| 155 | 4.06^*^ | 14.0, 2.1, 11.6^a^ | [75], [76] ,[77] |
| 10 | 3.99^*^ | 2.67 | [76] |
| 21 | 3.51^*^ | 3.08, 12.1^a^ | [76], [77] |
| 100 | 2.85^*^ | 36.9, 2.49, 3.8^a^ | [75], [76], [77] |
| 16 | 2.56 | 14.3, 4.8^a^ | [75], [77] |
| 301a | 1.96 | 34.2 | [75] |
| 199 | 1.81 | 2.46 | [76] |
| 34a | 1.71 | NR |  |
| 34c | 0.75 | NR |  |
| 29b | 0.22 | NR |  |
| 141 | -0.88 | -1.5 | [78] |
| 22 | -1.40 | NR |  |
| 148b | -1.81 | 0.31, -2.29 | [76], [78] |
| 210c | -2.18 | 2.97^b^, 2.82^b^ | [76], [78] |
| 212 | -2.70 | 22.2 | [75] |
| 148a | -2.81^*^ | 0.18, -3.3 | [76], [78] |
| 375 | -11.57^***^ | 0.46, -2.51 | [76], [78] |
