# Supplementary material for: Tissue and Serum microRNAs in the KrasG12D Transgenic Animal Model and in Patients with Pancreatic Cancer
Source: PLoS One. 2011 Jun 27;6(6):e20687. doi: 10.1371/journal.pone.0020687 (PMC3124473; doi:10.1371/journal.pone.0020687)
Supplement: Table S2 — Patient diagnoses and characteristics for the plasma sample set analyzed. (DOC) [file pone.0020687.s002.doc]

| **Group** | **Description** | **Age** | **Sex** | **T** | **N** | **M** | **stage** |
| --- | --- | --- | --- | --- | --- | --- | --- |
| **Pancreatic cancer** | malignant neoplasm of tail of pancreas | 85 | m | T3 | N0 | M0 | IIA |
|  | malignant neoplasm of head of pancreas | 72 | m | T3 | N1 | M0 | IIB |
|  | malignant neoplasm of pancreas, unspecified | 59 | m | T3 | N1 | M0 | IIB |
|  | malignant neoplasm of head of pancreas | 53 | f | T3 | N0 | M0 | IIA |
|  | malignant neoplasm of head of pancreas | 70 | m | T2 | N0 | M0 | IB |
|  | malignant neoplasm of tail of pancreas | 69 | m | T3 | N0 | M0 | IIA |
| **Non-cancer control** | chronic pancreatitis | 59 | f | n/a | n/a | n/a | n/a |
|  | cyst of pancreas | 48 | f | n/a | n/a | n/a | n/a |
|  | pseudocyst of pancreas | 68 | m | n/a | n/a | n/a | n/a |
|  | cyst of pancreas | 60 | f | n/a | n/a | n/a | n/a |
|  | benign neoplasm of endocrine pancreas | 54 | f | n/a | n/a | n/a | n/a |
|  | carcinoma in situ of breast | 53 | f | n/a | n/a | n/a | n/a |
|  | benign neoplasm of liver | 34 | f | n/a | n/a | n/a | n/a |
|  | cirrhosis of liver | 31 | m | n/a | n/a | n/a | n/a |
| **Upper GI cancer** | malignant neoplasm of duodenum | 59 | m | T3 | N0 | M0 | II |
|  | malignant neoplasm of lesser curvature of stomach | 49 | f | T2b | N1 | M0 | II |
|  | Gastrointestinal stromal tumor-stomach | 55 | m | n/a | n/a | n/a | n/a |
|  | malignant neoplasm of duodenum | 51 | f | T3 | N0 | M0 | II |
|  | malignant neoplasm of duodenum | 70 | m | T3 | N1 | M0 | III |
| **Colon cancer** | malignant neoplasm of ascending colon | 63 | m | T3 | N0 | M0 | IIA |
|  | benign neoplasm of transverse colon | 77 | m | n/a | n/a | n/a | n/a |
|  | malignant neoplasm of caecum | 93 | f | T2 | N0 | M0 | I |
|  | malignant neoplasm of rectum | 27 | f | n/a | N0 | M0 | n/a |
|  | malignant neoplasm of rectum | 83 | f | T3 | N1 | M0 | III B |
| **Liver cancer** | secondary malignant neoplasm of liver, unknown origin | 58 | f | n/a | n/a | M1 | IV |
|  | Liver adenocarcinoma | 50 | f | n/a | n/a | M0 | n/a |
|  | Hepatocellular carcinoma | 56 | m | T1 | N0 | M0 | I |
