# Supplementary material for: Tissue and Serum microRNAs in the KrasG12D Transgenic Animal Model and in Patients with Pancreatic Cancer
Source: PLoS One. 2011 Jun 27;6(6):e20687. doi: 10.1371/journal.pone.0020687 (PMC3124473; doi:10.1371/journal.pone.0020687)
Supplement: Table S3 — mRNA expression of miR target genes in pancreatic adenocarcinoma. mRNA expression levels were obtained from published studies that compared normal and cancer tissues. Targets for those miRs found upregulated in the circulation of pancreatic cancer patients were derived from an in silico analysis (see Methods). n, number of samples per group. * p<0.05, ** p<0.01, *** p<0.001 downregulation in cancer versus control tissues. (DOC) [file pone.0020687.s003.doc]

|  | Reference #: | [79] | [80] |
| --- | --- | --- | --- |
|  | Control (n)  Cancer (n) | 10  10 | 6  8 |
| miR | Target gene | Downregulation of mRNA | |
| 10a | EVI1 | * | *** |
|  | FGFR1 | *** |  |
|  | GPC3 | *** |  |
| 21 | BCL2 | * |  |
| 223 | PSIP1 |  | *** |
| 155 | PSIP1 |  | *** |
|  | MLH1 | *** |  |
| 221 | PTEN | *** | ** |
